# Antineutrophil Cytoplasmic Autoantibody (ANCA)-Associated Vasculitis With Mucosal Involvement Following COVID-19 Pneumonia

**DOI:** 10.7759/cureus.31441

**Published:** 2022-11-13

**Authors:** Hoang Ta, Hussein Awada, Puneet Kang, Nathaniel Gilbert, Nairmeen Haller, Eliot Mostow, Jason Lane, Inderprit Singh

**Affiliations:** 1 Internal Medicine, Cleveland Clinic Akron General, Akron, USA; 2 Tranlational Hematology and Oncology, Cleveland Clinic, Cleveland, USA; 3 Internal Medicine, University Hospitals Cleveland Medical Center, Cleveland, USA; 4 Research, Cleveland Clinic Akron General, Akron, USA; 5 Dermatology, Cleveland Clinic Akron General, Akron, USA; 6 Pathology, Cleveland Clinic Akron General, Akron, USA; 7 Rheumatology, Cleveland Clinic Akron General, Akron, USA

**Keywords:** hemorrhagic bullae, neutrophil extracellular traps, superantigens, pauci-immune necrotizing crescentic glomerulonephritis, leukocytoclastic vasculitis, post-covid-19 inflammatory response, anca-associated vasculitides

## Abstract

Antineutrophil cytoplasmic autoantibody (ANCA)-associated vasculitides (AAV) are a group of inflammatory disorders in which autoantibodies damage small arteries throughout the body, including in the upper and lower respiratory system, kidneys, as well as the skin. AAV may be precipitated by a variety of causes, including infections. In this report, we examine the case of a patient who developed AAV that was suspected primarily based on mucocutaneous hemorrhagic bullae, elevated ANCA levels, and subsequently confirmed by kidney biopsy, while recovering from coronavirus disease 2019 (COVID-19) infection. AAV and COVID-19 infections may present with similar symptoms, rendering an accurate diagnosis challenging. Additionally, only a few other cases describing a similar onset of AAV post-COVID-19 infection have been described in the literature. Initial presenting features of AAV in such cases have varied considerably, which makes the diagnosis even more challenging. We also engage in a review of such cases to assess key similarities, different treatment options, and outcomes. Lastly, the fact that several mechanisms have been proposed for AAV highlights the need for continued research to help clarify the pathophysiology while also identifying the optimal therapy.

## Introduction

Antineutrophil cytoplasmic autoantibody (ANCA)-associated vasculitides (AAV) are a group of rare autoimmune inflammatory disorders that involve self-reactive autoantibodies resulting in inflammation and damage to small and medium-sized arteries. The spectrum of AAV includes granulomatosis with polyangiitis (GPA), microscopic polyangiitis (MPA) as well as eosinophilic granulomatosis with polyangiitis (EGPA), each of which is defined by its own characteristic, yet diverse, clinical features. The hallmark of all of these disorders is necrotizing vasculitis, which typically results in multiorgan system failure. While AAV may affect various organs, it mainly involves the upper and lower respiratory tracts, kidneys, and skin. While GPA and EGPA are primarily associated with anti-proteinase autoantibodies (PR3-ANCA, also referred to as c-ANCA), MPA is more commonly characterized by positive perinuclear autoantibodies (MPO-ANCA, also known as p-ANCA) [1]. Less commonly, patients with these disorders may present with the opposite serology or may be seronegative [1,2]. While some patients may have a genetic predisposition, many AAV patients experience disease onset following inflammatory environmental and infectious triggers [3]. In the current coronavirus disease 2019 (COVID-19) pandemic, reports have been emerging of cases of new-onset autoimmune disorders triggered by COVID-19 immune provocation [4]. We present a case of a 67-year-old male who developed a post-COVID-19 inflammatory response with AAV multiorgan involvement, including the first documented instance of mucocutaneous hemorrhagic bullae.

## Case presentation

**Figure 1:**
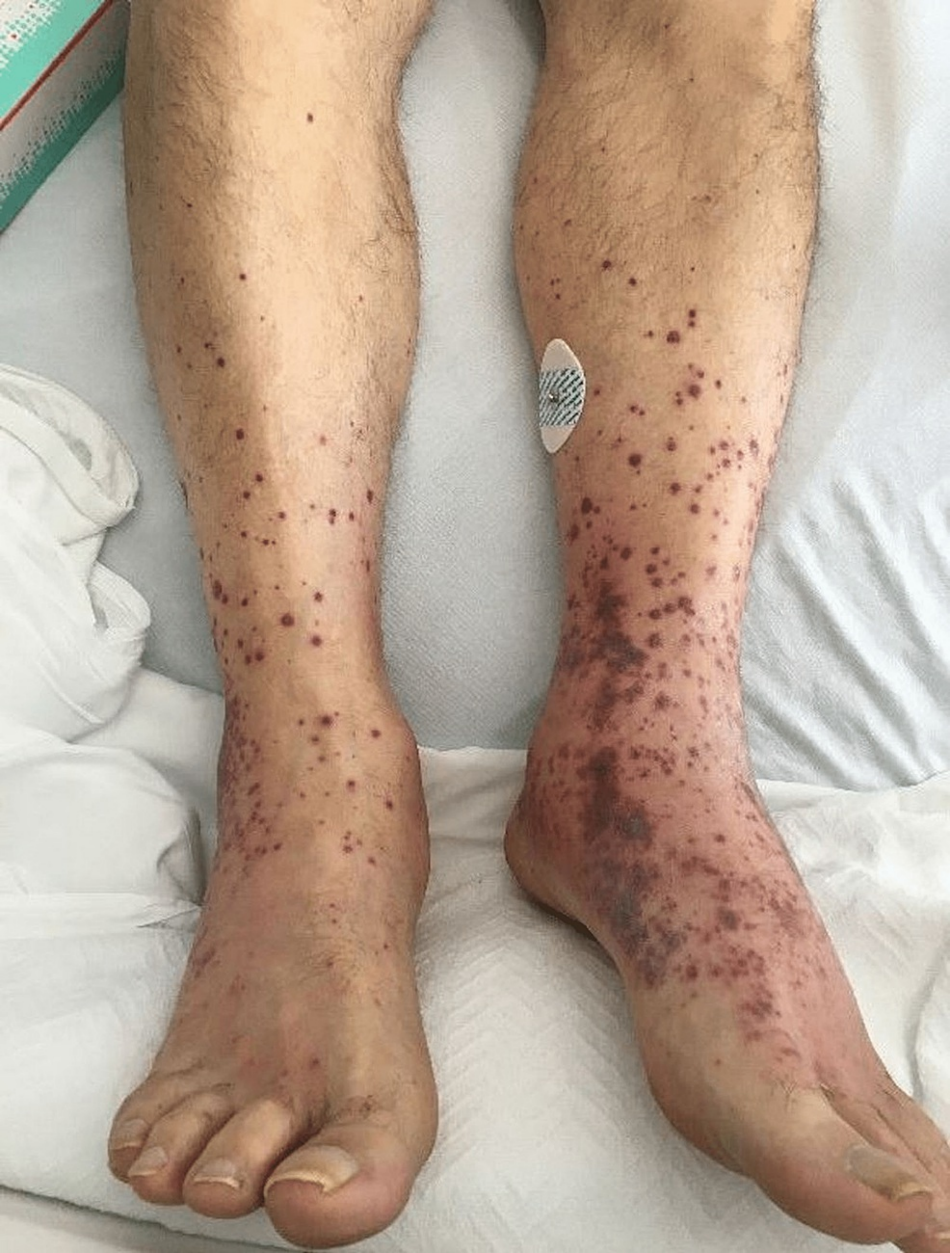
Initial presentation of the diffuse purpuric rash of the lower legs

**Figure 2:**
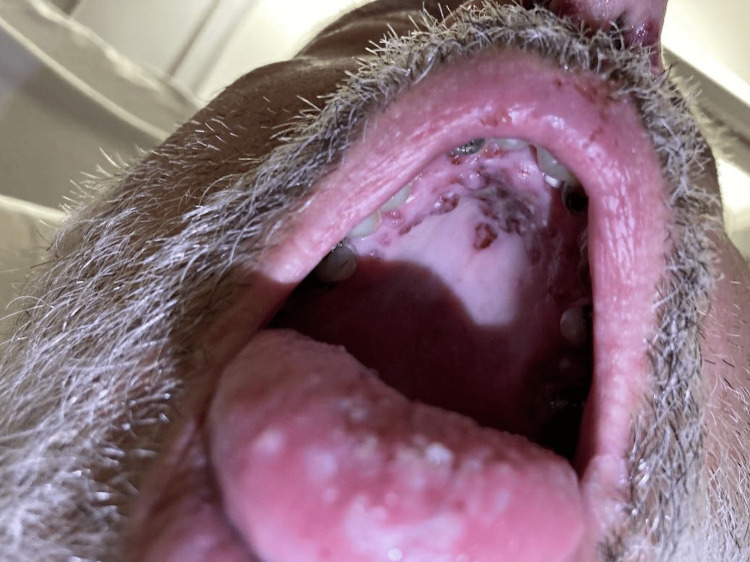
Oral lesions of the hard palate (hospitalization day one)

A 67-year-old previously healthy Caucasian male with a past medical history confined to chronic back pain presented to the emergency department (ED) with the chief complaints of shortness of breath, epistasis, hemoptysis, and a diffuse purpuric rash (Figure 1). Preceding ED visit, he had recently been hospitalized two weeks prior for a COVID-19 infection with superimposed bacterial pneumonia that had been treated with ampicillin-sulbactam and then azithromycin on discharge. In the interim, he had been seen by his primary care provider, who had prescribed codeine for cough suppression. The patient had a history of intermittent codeine use for back pain, but he did not use any other medications. In an attempt to suppress his cough, he had completed an entire bottle of codeine prior to going to bed. He had woken up the following morning to find the emergence of the rash (Figure 1). He described the rash as small, red, pruritic, painful lesions that had first appeared on his legs and had since extended to his hands, back, and abdomen. He also endorsed painful oral sores that had started during his last hospitalization, which had led to a 15 lbs weight loss, though he did not have odynophagia, dysphagia, drooling, or emesis (Figure 2).

**Figure 3:**
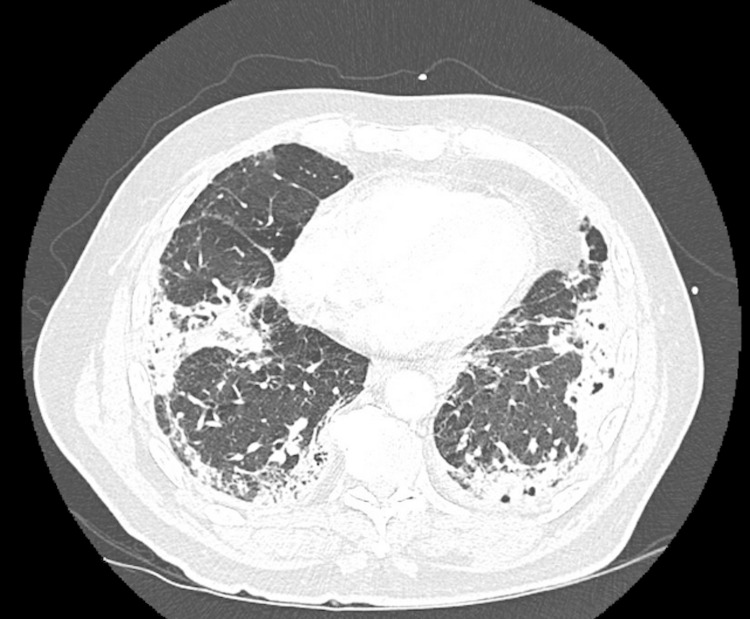
CT chest CT chest showed multiple predominantly peripheral areas of consolidation in both lungs. The consolidation appeared dense with cystic areas within the consolidation, raising concern for necrotizing pneumonia CT: computed tomography

The patient appeared tachypneic and in distress on examination. He was found to be in acute hypoxic respiratory failure requiring 4 liters of supplemental oxygen. Initial laboratory workup revealed a white blood count of 14.4 k/uL (normal range: 3.7-11 k/uL), lactate of 2.5 mmol/L (0.4-2.0 mmol/L), procalcitonin of 1.02 ng/mL (<0.09 ng/mL), erythrocyte sedimentation rate (ESR) of 102 mm/hr (0-15 mm/hr), and C-reactive protein (CRP) of 25 mg/dL (<0.9 mg/dL). Urine studies showed an albumin/creatinine ratio of 93 mg/g (<30 mg/g), proteinuria of 100 mg/dL (<0.0 mg/dL), and urine red blood cells of 3-5/HPF (0-3/HPF) with large hemoglobinuria. COVID-19 PCR x2 were negative. CT chest showed extensive bilateral cystic consolidations of pulmonary peripheral infiltrates concerning for necrotizing pneumonia (Figure 3), along with enlarged mediastinal and hilar lymphadenopathy, and splenomegaly. His clinical presentation was suggestive of an inflammatory process with a possible autoimmune component rather than infectious or malignant processes. He was given morphine, oxycodone, diphenhydramine, oral prednisone 40 mg, vancomycin, and Zosyn. He was admitted to the hospital for further management.

**Figure 4:**
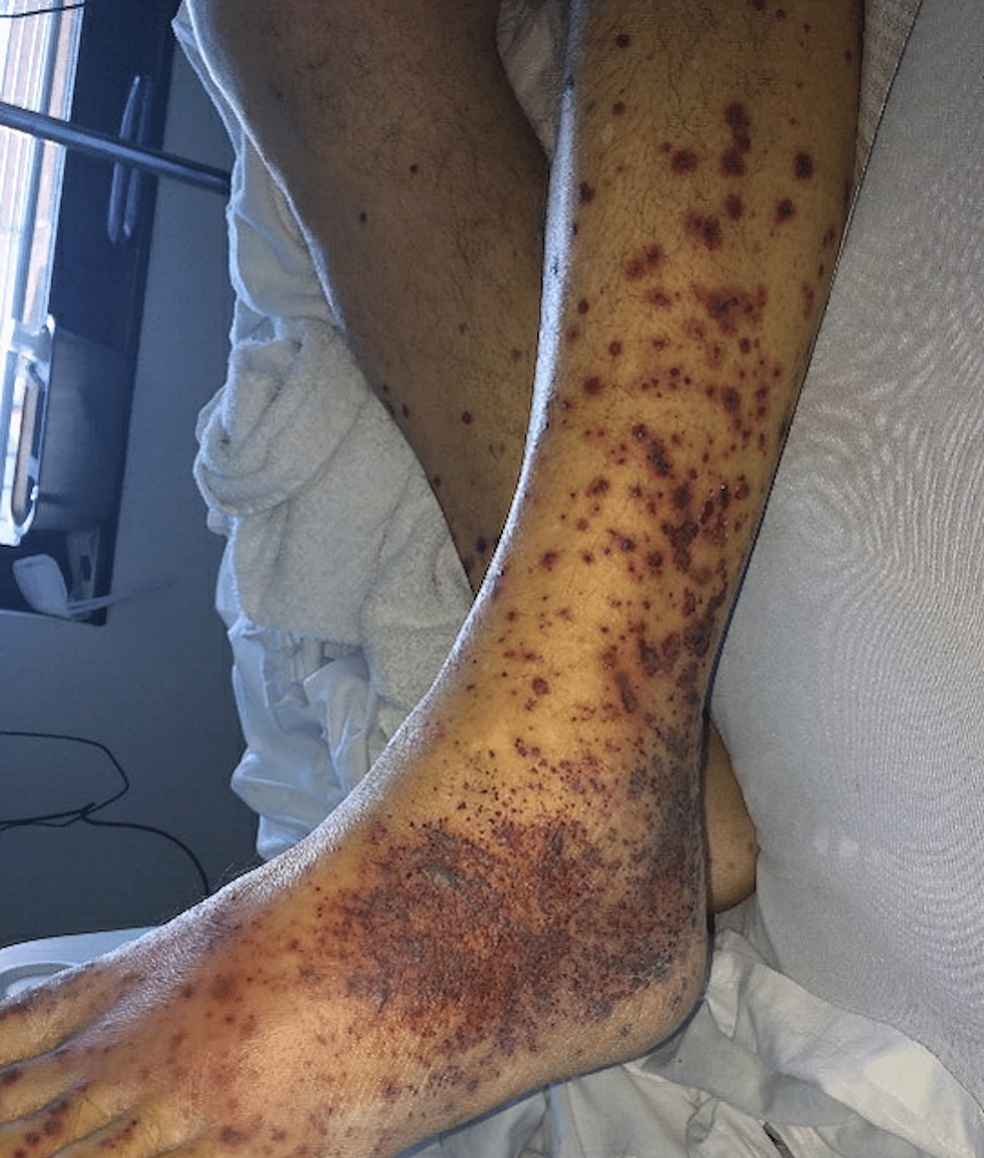
Worsening skin manifestations of papular rash with hemorrhagic vesicles (hospitalization day four)

**Figure 5:**
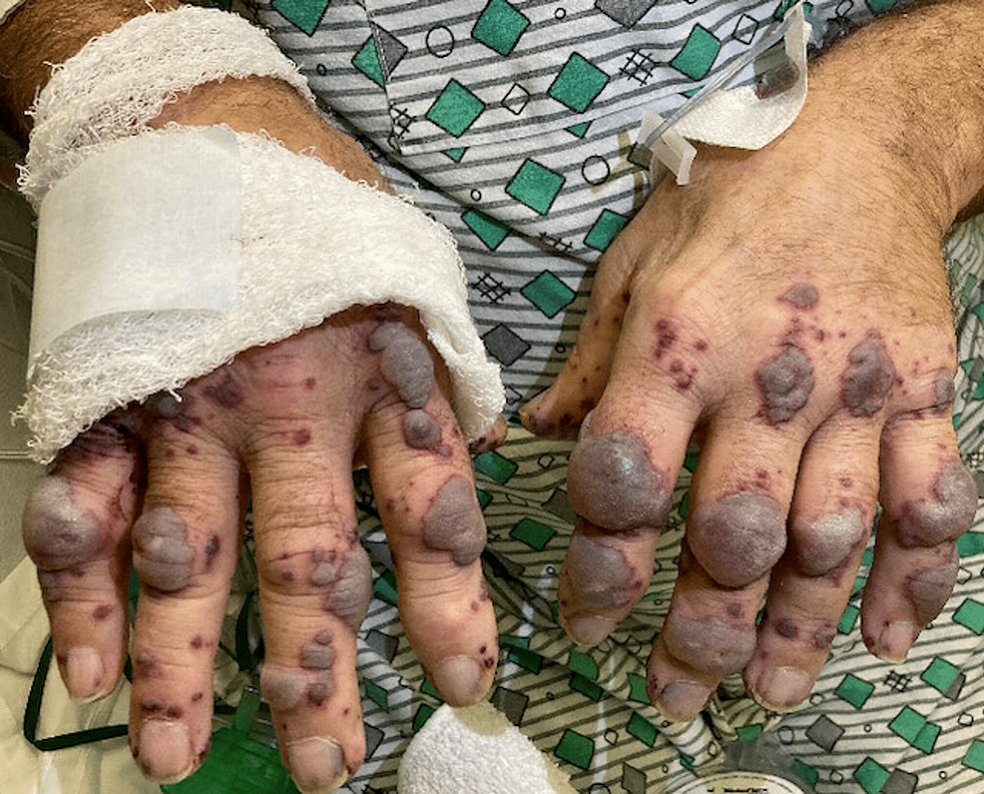
Progression to hemorrhagic bullae of the hands (hospitalization day four)

**Figure 6:**
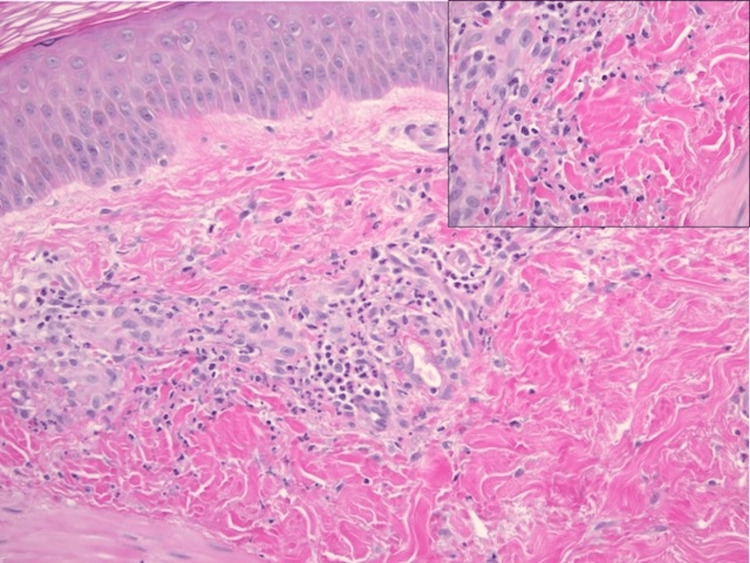
Hematoxylin and eosin (H&E)-stained biopsy H&E-stained section of biopsy of the skin of the right leg showing perivascular and interstitial neutrophils (200x magnification), with the inset highlighting scattered extravasated erythrocytes and leukocytoclasis (600x magnification), overall findings consistent with early or mild leukocytoclastic vasculitis

During the course of hospitalization, the patient experienced worsening skin manifestations of the hemorrhagic bullae (Figures 4, 5), affecting hand movements, and leading to hematuria, hoarseness, severe oral and body pain along with a reported “sandy” sensation in the eyes. Because of worsening symptoms raising suspicions for vasculitis, rheumatology, infectious disease, and nephrology were consulted. The patient was started on pulse dose steroids with IV Solu-Medrol 1000 mg for three days. His autoimmune workup was positive for c-ANCA (>8.0 AI, normal: <1.0), indicative of ANCA vasculitis; it also revealed anticardiolipin antibody of 53 MPL (normal: <12.5), antinuclear antibodies of 1:160, IgG of 1270 mg/L, IgA of 339 mg/L, as well as cryoglobulin IgG and IgM at 2 mg/dL and 3 mg/dL, respectively. C4 was low at 7 mg/dL (normal: 13-46) while C3 was normal. Negative serology included anti-SSA, anti-SSB, anti-dsDNA, anti-RNP, anti-b-2 glycoprotein, anti-histone, and anti-smooth-muscle. Both ferritin and triglycerides were elevated at 3,479 ng/mL (normal: 30.2-565.7) and 197 mg/dL (normal: <150), respectively. Infectious workup included blood, wound, and urine cultures, viral hepatitis panel, HIV, Bartonella, and COVID-19 PCR x2, all of which were negative. A skin punch biopsy of the lower extremities demonstrated perivascular and interstitial neutrophils and rare eosinophils, with scattered extravasated erythrocytes consistent with leukocytoclastic vasculitis (Figure 6). The skin biopsy results were nonspecific. Furthermore, a renal biopsy was pursued because of the new onset of proteinuria with an unexplained elevation of urine albumin/creatinine ratio, and a preserved kidney function with a creatinine level of 0.9. The renal biopsy revealed a pauci-immune necrotizing crescentic glomerulonephritis consistent with AAV. The patient responded well to pulse dose steroids; his breathing improved, and most of the bullae on his hands ruptured. The patient's main symptoms were dermatological and not life-threatening, and hence rituximab or cyclophosphamide was not necessary. He was discharged on a steroid taper, sulfamethoxazole, and trimethoprim. His six-week post-hospitalization follow-up showed complete resolution of bullae with well-healed scabs and purple-pigmented patches. As such, the patient responded well to glucocorticoid monotherapy, and hence rituximab and cyclophosphamide were not initiated by rheumatology.

## Discussion

A comprehensive search of the literature yielded 11 studies that mentioned the association between AAV and post-COVID-19 infection on Pubmed, Embase, and Google Scholar databases between 2020-2021. We presented our case of COVID-19 hyperimmune stimulation-induced ANCA-related vasculitis and analyzed its association with the 11 studies (Table 1). Our case involves a previously healthy male who developed AAV that was likely triggered by post-COVID-19 hyperinflammatory syndrome as suggested by the continuously elevated ferritin and triglycerides. Only a few case reports in the literature have described the incidence of AAV following COVID-19 infection, and none of the reported cases share similar cutaneous manifestations of AAV in the form of hemorrhagic bullae as in our patient [5-13]. In addition, none of the reported cases had oral mucosal involvement. Skin involvement usually occurs in 30-50% of patients with AAV, and it usually presents as purpura of the lower extremities but can also manifest as livedo reticularis, urticaria, or nodules along with focal necrosis and ulceration [14]. Nevertheless, hemorrhagic bullae are not known to be associated with AAV but rather with other types of vasculitis such as Henoch-Schönlein purpura (HSP). Indeed, only four cases in the literature have described hemorrhagic bullae in EGPA, none of which were related to COVID-19 infection [15-18]. The lack of significant eosinophilic infiltration in our patient rules out EGPA and in turn distinguishes this case from those in the literature. Moreover, the absence of similar reported presentations brings into question whether these lesions would best be treated with aspiration, or puncturing, in addition to whether the patient would require wound care or be at risk of infection in the setting of steroid therapy. In our case, a few of the bullae burst while the majority resolved spontaneously without any additional intervention.

**Table 1:** ANCA-related vasculitis following COVID-19 infection Previous cases of ANCA-related vasculitis following COVID-19 infection between 2020 and 2021. Demographics and organ system involvement are included to highlight the variety in clinical presentation between cases ANCA: antineutrophil cytoplasmic autoantibody; COVID-19: coronavirus disease 2019; LAD: lymphadenopathy; GN: glomerulonephritis; GGOs: ground glass opacities; BAL: bronchoalveolar lavage

Case	Age (years)	Sex	Lung involvement	Skin involvement	Mucosal involvement	Kidney pathology
Our case	67	Male	Cavitary lesions concerning necrotizing pneumonia. Mediastinal and hilar LAD	Leukocytoclastic vasculitis	Oral and nasal	Necrotizing crescentic GN
Izci Duran et al., 2021 [5]	36	Female	GGOs indicating bilateral cavitary lesions	None	None	Necrotizing crescentic GN (pauci-immune)
Uppal et al., 2020 [6]	46	Male	Unknown	Leukocytoclastic vasculitis	None	Necrotizing GN (pauci-immune)
Moeinzadeh et al., 2020 [7]	25	Male	Bilateral GGOs resembling alveolar hemorrhage	None	None	Crescentic proliferative GN
Hussein et al., 2020 [8]	37	Female	GGOs resembling alveolar hemorrhage	None	None	N/A
Maritati et al., 2021 [9]	64	Female	Interstitial GGOs, BAL negative for alveolar hemorrhage	None	None	Necrotizing crescentic GN (pauci-immune)
Izci Duran et al., 2021 [5]	26	Male	GGOs indicating alveolar hemorrhage	None	None	Crescentic GN (pauci-immune)
Uppal et al., 2020 [6]	64	Male	CXR showing bilateral patchy opacities	None	None	Crescentic GN (pauci-immune)
Madanchi et al., 2021 [10]	53	Male	Bilateral patchy basilar opacities; alveolar hemorrhage on BAL	None	None	Crescentic GN (pauci-immune)
Allena et al., 2021 [11]	60	Female	Crazy paving pattern; alveolar hemorrhage on BAL	None	None	Crescentic GN (pauci-immune)
Cobilinschi et al., 2021 [12]	67	Female	GGOs	Necrosis and neutrophil infiltrates; MRSA-positive	None	N/A
Wali et al., 2021 [13]	26	Female	GGOs, suspicion for alveolar hemorrhage	None	None	Crescentic GN (fine, granular IgG fluorescence)

Besides cutaneous symptoms, AAV causes upper or lower respiratory necrotizing damage in 90% of patients as compared to only 18% of the patients showing evident glomerulonephritis at presentation [19-20]. While such multisystem involvement typically raises suspicion for an ongoing autoimmune process, this may instead be challenging in the current pandemic as many of the AAV symptoms are either shared with COVID-19 or expected from COVID-19-related complications. This is particularly tricky since the majority of AAV patients present with upper and lower respiratory symptoms that may mimic COVID-19 infection before another organ system becomes more evidently involved. In addition, the enormous current volume of COVID-19 patients compared to GPA may elicit availability biases in physicians and increase the risk of missing out on alternative, though less common, differential diagnoses. Moreover, the lack of cases in the literature makes it even harder to characterize AAV post-COVID-19 as compared to other AAV cases. Indeed, our case is the only one to report mucosal involvement in the literature, and this further increases the ambiguity regarding the likelihood of mucosal and other organ involvement in post-COVID-19 AAV compared to traditional AAV.

A total of 12 patients (including ours) with the post-COVID-19 onset of AAV have been reported. Six of the 12 patients have been men (50%), and the mean age at diagnosis was 47.58 years. Six of them were positive for c-ANCA while the other six had p-ANCA-positive serology. All patients had lung disease, and all but two had kidney disease. Only three patients had skin pathology. Mucosal involvement and cutaneous hemorrhagic bullae were present only in our patient. Three patients received conventional COVID-19 therapy, while three were treated with the antiviral Favipravir, one patient received COVID-19 monoclonal immunoglobulins, and another patient was treated with tocilizumab and convalescent plasma. Four of the patients ended up requiring renal replacement therapy. All but one of the patients have survived beyond the COVID-19 infection phase. AAV treatment included glucocorticoids in all patients, while seven patients were treated with cyclophosphamide, six patients received rituximab (two of whom were also receiving cyclophosphamide), one patient underwent plasmapheresis, and another had plasma exchange.

Though COVID-19 has been reported to trigger autoimmune disorders, the mechanism through which the pathophysiology is driven remains unclear [21]. That said, limited evidence exists in the literature to explain the mechanisms by which infectious insults induce the autoimmune response underlying the pathophysiology of AAV. Previous studies have suggested Staphylococcus aureus-triggered AAV being mediated by superantigens, which prompt the hyperactivation of the immune system by inciting a non-specific polyclonal T-cell activation and subsequent massive cytokine release [22]. Similarly, studies have been emerging to emphasize the superantigenic role of the severe acute respiratory syndrome coronavirus 2 (SARS-CoV-2) in evoking post-COVID-19 hyperinflammatory states [23-25]. This may have further been potentiated by the use of codeine in our patient, which in turn activates human mast cell degranulation and the production of proinflammatory chemokines [26]. While the patient previously tolerated codeine, we speculate that an anaphylactoid reaction to excessive doses may have contributed to a dermatological presentation similar to HSP.

Other mechanisms through which COVID-19 may elicit autoimmunity include molecular mimicry, viral persistence, epitope spreading as well as the formation of neutrophil extracellular traps (NET) [27,28]. NET are net-like structures that consist of DNA-histone complexes and proinflammatory proteins released by activated neutrophils. They contribute to the development of AAV through endothelial injury and complement activation while promoting the production of c-ANCA and p-ANCA [29]. The expression of these NET has been demonstrated to be increased in COVID-19 patients [30]. Nevertheless, the scarcity of reported AAV cases post-COVID-19 hinders the challenge of unmasking the exact mechanism provoking its onset. This, in turn, highlights the importance of our case and signals the need to report more cases that would help elucidate the disease process.

## Conclusions

We presented a case of COVID-19 hyperimmune stimulation and AAV, a case that highlights issues associated with diagnosing new-onset AAV, which can be challenging during the current COVID-19 pandemic. The key features that would hint towards the autoimmune nature of patients’ presentation may be skin involvement and rapidly progressive kidney disease irrespective of the severity of the pulmonary damage. However, many patients lack these features on presentation and may end up being overlooked. In addition, some patients, such as ours, may have unusual skin manifestations that relate to AAV. Future studies should focus on elucidating the role of SARS-CoV-2 in triggering autoimmune insults and evoking characteristic manifestations. Recommendations should also be made with the aim of identifying ANCA vasculitis in this setting early on in order to prevent irreversible multiorgan damage.
